# Photodegradation and van der Waals Passivation of Violet Phosphorus

**DOI:** 10.3390/nano14050422

**Published:** 2024-02-25

**Authors:** Xiangzhe Zhang, Bowen Lv, Haitao Wei, Xingheng Yan, Gang Peng, Shiqiao Qin

**Affiliations:** 1 College of Advanced Interdisciplinary Studies, National University of Defense Technology, Changsha 410073, China; xiangzhe.cheung@gmail.com (X.Z.); lvbowen01@163.com (B.L.); Weihaitao0903@163.com (H.W.); 2 College of Aerospace Science and Engineering, National University of Defense Technology, Changsha 410073, China; 17801016168@163.com; 3 College of Science, National University of Defense Technology, Changsha 410073, China

**Keywords:** violet phosphorus, photodegradation, van der Waals passivation

## Abstract

Violet phosphorus (VP), a novel two-dimensional (2D) nanomaterial, boasts structural anisotropy, a tunable optical bandgap, and superior thermal stability compared with its allotropes. Its multifunctionality has sparked widespread interest in the community. Yet, the VP’s air susceptibility impedes both probing its intrinsic features and device integration, thus making it of urgent significance to unveil the degradation mechanism. Herein, we conduct a comprehensive study of photoactivated degradation effects on VP. A nitrogen annealing method is presented for the effective elimination of surface adsorbates from VP, as evidenced by a giant surface-roughness improvement from 65.639 nm to 7.09 nm, enabling direct observation of the intrinsic morphology changes induced by photodegradation. Laser illumination demonstrates a significant thickness-thinning effect on VP, manifested in the remarkable morphological changes and the 73% quenching of PL intensity within 160 s, implying its great potential for the efficient selected-area etching of VP at high resolution. Furthermore, van der Waals passivation of VP using 2D hexagonal boron nitride (hBN) was achieved. The hBN-passivated channel exhibited improved surface roughness (0.512 nm), reduced photocurrent hysteresis, and lower responsivity (0.11 A/W @ 450 nm; 2 μW), effectively excluding adsorbate-induced electrical and optoelectrical effects while disabling photodegradation. Based on our experimental results, we conclude that three possible factors contribute to the photodegradation of VP: illumination with photon energy higher than the bandgap, adsorbed H_2_O, and adsorbed O_2_.

## 1. Introduction

Phosphorus allotropes, as exemplified by white, red, black, and violet phosphorus, offer a rich palette of physical properties within their large phase diagram [1]. Yet, the air instability and the toxicity of white phosphorus [1,2], and the low conductivity of red phosphorus [3,4] hinder their widespread exploration and utilization in electronics. In comparison, the two-dimensional (2D) black (BP) and violet phosphorus (VP, encompassing VP_11_ [5] and VP_21_ [6]) have recently garnered significant attention due to their appealing properties, such as good thermal stability [6,7], in-plane anisotropy [6,8,9], tunable direct bandgaps [7,10,11,12], substantial carrier mobilities [7,13], and associated promising optoelectronic prospects [12,14,15,16,17], which make them ideal platforms for both fundamental physics and application researches. Most of all, VP’s reported superior thermal stability [6,18] to other allotropes [10,19,20] positions it as a promising alternative that meets the pressing need for a more robust material. However, there exists a major hurdle ahead of the practical implementation and integration of VP in optoelectronics. Previous studies have reported experimentally observing the degradation of VP in ambient conditions [5,6,12,18,21], which is detrimental to understanding the intrinsic nature of VP and complicates the fabrication of VP devices. Analogous to its allotrope BP [19,22,23,24,25], much of the related literature ascribe the degradation to VP’s surface hydrophilicity [1,19,23,25,26,27] leading to the substantial adsorption of H_2_O/O_2_, while few works, except a recent one by Ghafariasl [28], have focused on the impact from light illumination. Moreover, research on the passivation of VP is currently lacking, and facile and effective pathways to preserve or restore VP’s intrinsic surface are also urgently sought.

For this study, to unravel the underlying mechanism of VP degradation and address the limitations mentioned above, we systematically investigated the degradation effect of mechanically exfoliated VP (VP_21_ [6] is used in our work) flakes through morphological, spectroscopy, electrical, and optoelectrical characterization. Our findings revealed that laser illumination substantially accelerates the degradation of VP. Following thermal annealing in a flowing inert N_2_ environment, the adsorbate film on the photodegraded VP surface was effectively eliminated, allowing direct observation of the intrinsic morphological features at the photodegraded points. Additionally, atomic-force microscopy (AFM) and scanning electron microscopy (SEM) characterization indicated a notable etching effect on VP induced by laser illumination. This phenomenon was also supported by Raman and photoluminescence spectra, which exhibited illumination-duration-dependence, implying its great potential for efficient and high-resolution selected-area etching of VP. Moreover, by employing 2D hexagonal boron nitride (hBN) as the passivation layer, van der Waals passivation of VP is achieved. AFM analysis confirmed that the hBN passivation successfully excluded adsorbates and prevented photodegradation, from which we can conclude that, without the presence of adsorbates, light illumination alone fails to stimulate the degradation. After being integrated into a photodetector, compared with the bare channel, the hBN-covered channel exhibited lower photocurrent hysteresis and a one-stage, fast photoresponse. These phenomena originate from the reduced adsorbate-induced trap states, highlighting an excellent passivation effect. Finally, based on our experimental results, we attribute the photo-accelerated degradation of VP to a combination of three possible factors: photon energy exceeding the bandgap, adsorbed H_2_O, and adsorbed O_2_.

## 2. Materials and Methods

### 2.1. Sample Preparation

In this work, we utilized VP and hBN crystals synthesized via the chemical-vapor transport method and provided by Taizhou SUNANO New Energy Co., Ltd. (Taizhou, China) To be specific, the VP crystals used in our work belong to the monoclinic space group P2/n, with unit-cell constants of a = 9.210 Å, b = 9.128 Å, c = 21.893 Å, and β = 97.776°, also referred to as VP_21_ [5,6]. Before exfoliation, the bulk crystals are stored in an inert N_2_ atmosphere and kept in darkness to prevent degradation. The VP and hBN flake samples in our work were obtained by mechanical exfoliation from the bulk crystals onto silicon oxide substrates (300 nm SiO_2_) using ELP BT-130E-SL tapes (Nitto, Osaka, Japan), excluding the solution-induced effects from liquid-phase exfoliation methods [21,22]. To stack up the hBN/VP heterostructures, we adopted a PMMA-mediated transfer technique. Initially, in a glove box, an hBN flake and a VP flake with the desired geometries were pre-exfoliated onto two individual silicon oxide substrates. Then, with the aid of a homemade transfer system in the glove box, a glass slide/PDMS/PMMA handler was used to delicately pick up the hBN flake from its parent substrate. After that, the hBN flake, now adhered to the handler, was aligned and carefully brought into contact with the pre-exfoliated VP flake under an optical microscope. To facilitate the transfer, the substrate was warmed to 80 °C, enabling the hBN flake to detach from the PMMA film and adhere onto the VP flake. Following the transfer, the hBN/VP heterostructure sample was first coated with AR-P 672.06 PMMA (ALLRESIST GmbH, Strausberg, Germany) inside the glove box and then spin-coated at a rotation speed of 4000 rpm for 60 s, resulting in a uniform PMMA layer with a thickness of approximately 450 nm. Subsequently, the coated samples were introduced into an electron-beam lithography system (RAITH GmbH, Dortmund, Germany) for electrode patterning. Cr/Au (5 nm/70 nm) electrodes were then constructed on the patterned samples via electron-beam evaporation (TECHNOL, Beijing, China). Upon completion of the lift-off process, the as-fabricated VP devices were promptly placed back into the glove box to avoid air exposure.

### 2.2. Characterization and Measurement

All bright-field and dark-field optical micrographs were captured using a ZEISS Axio optical microscope (ZEISS, Shanghai, China). The morphological details of the samples were characterized by an NT-MDT Prima AFM system (Apeldoorn, Netherlands) operating in semi-contact mode and a Hitachi SEM system with operation parameters of 10 kV and 10 μA. Raman and photoluminescence spectra were measured through a WITec Alpha300R confocal Raman system (WITec, Ulm, Germany) equipped with a 100X objective lens (NA: 0.95) and excited by a 532 nm laser. To prevent photodegradation during measurement, we used a laser power of 100 μW and limited the integration time to 1 s. For electrical transport and photoresponse measurements, the VP devices were tested inside a Cindbest probe-station system (Cindbest, Shenzhen, China) with a vacuum sample chamber (10^−4^ Pa). A Keithley 2450 source meter was used to apply the electrical voltage and examine the current signals, while a Zolix TLS3-X300P-G xenon lamp (Zolix, Beijing, China) served as the illumination source. To determine the elemental concentration of bulk VP crystals in different states, we performed XPS analysis using a Thermo ESCALAB 250Xi X-ray photo-electron spectroscopy system (Thermo Fisher, Waltham, MA, USA); the peak of adventitious carbon (284.8 eV) was used as the calibration reference for accurate elemental quantification.

## 3. Results

**Figure 1:**
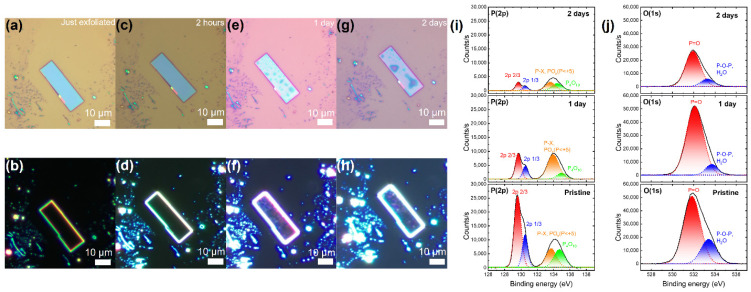
Degradation of VP flakes in ambient conditions. (**a**–**h**) The degradation process of an exfoliated VP flake after various air exposure times. (**a**,**b**) Bright-field (BF) and dark-field (DF) optical micrographs of the just exfoliated VP flake. (**c**,**d**) BF and DF optical micrographs of the VP flake after 2 h of air exposure, (**e**,**f**) after 1 day, and (**g**,**h**) after 2 days. (**i**,**j**) XPS P(2p) and O(1s) cores of a bulk VP crystal. The panels (from bottom to top) show the XPS spectra for the VP crystal in the pristine state, after 1 day of air exposure, and after 2 days of exposure, respectively.

Figure 1a,b present a VP flake freshly exfoliated onto a silicon dioxide substrate (300 nm SiO_2_). In the bright-field (BF) and dark-field (DF) optical micrographs (OMs), the freshly exfoliated VP flake surface appears clean and uniform, devoid of noticeable adsorbates. According to the BF and DF OM images in Figure 1c,d, there appear to be no significant morphological alterations on the VP surface within the initial two-hour exposure to room conditions (25 °C, 50% humidity). Nevertheless, after exceeding 3 h of exposure (detailed in Figure 1e–h and Figure S1), droplets begin to emerge on the VP surface and progressively aggregate into larger formations over time. Simultaneously, the color of the VP flake transits from blue (just exfoliated; Figure S1a) to pink (8 days; Figure S1l), demonstrating a discernible degradation process. To further investigate the morphological changes during the degradation process, SEM was carried out on another VP flake shown in Figure S2, from which a similar degradation process was observed more clearly.

To comprehend the degradation mechanisms of VP in ambient conditions, we performed an XPS analysis on a bulk VP crystal at various exposure durations in ambient conditions. The results are presented in Figure 1i,j (from bottom to top) for the XPS spectra of the VP crystal in the pristine state, after 1 day of exposure, and after 2 days of exposure in ambient conditions (without light illumination), respectively. Figure 1i demonstrates the P(2p) cores of the VP crystal; the spectra can be deconvoluted using Gaussian fitting into 4 subsidiary peaks, namely 2p_3/2_ (red), 2p_1/2_ (blue), P-X (P < +5, orange), and P_4_O_10_ (green), located at 129.6, 130.5, 133.7, and 134.7 eV, respectively [12,25,29,30,31,32]. In contrast, the O(1s) core spectra displayed by Figure 1j can be fitted into two subsidiary peaks located at 532.1 eV (red) and 533.7 eV (blue), which originate from dangling oxygen (P=O) and bridging oxygen (P-O-P or H_2_O), respectively [12,25,30,31,32,33,34,35,36]. The presence of these oxygen species indicates the interaction of VP with ambient oxygen, potentially leading to degradation. In the pristine state, since the VP undergoes a short air exposure before being loaded into the XPS chamber, both elemental P^0^ (including 2p_3/2_ and 2p_1/2_) and phosphate species coexist in the VP crystal. The P^0^ peak is more pronounced than the phosphate-species peak for the pristine state. With increasing air-exposure duration, as shown in the middle and top panels of Figure 1i, the phosphate-species peak becomes more prominent. According to the elemental-concentration variations derived from the XPS data presented in Table S1, the share of P decreases from 45.03% (pristine) to 33.18% after 1 day of exposure, and further to 29.94% after 2 days of exposure. Conversely, the share of O rises from 54.97% (pristine) to 66.82% (1 day), and finally to 70.06% (2 days). These observations suggest that the degradation of VP in ambient conditions stems from the hydrophilic nature of phosphorus, which is favorable for H_2_O/O_2_ adsorption and subsequent reaction with the VP to form phosphorus oxide (PO_x_) on the VP surface.

**Figure 2:**
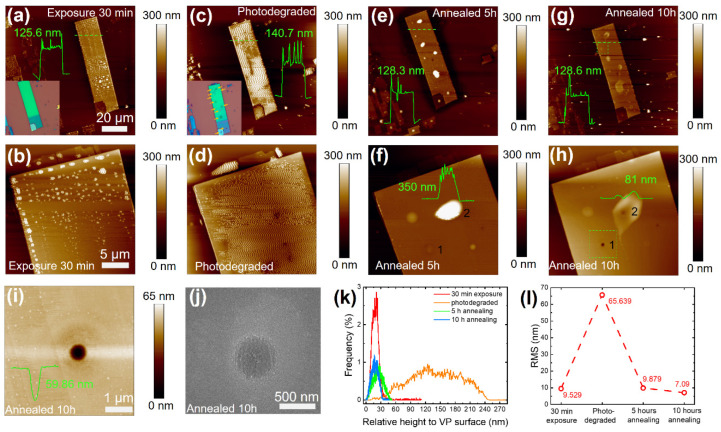
Morphological characterization of VP photodegradation. (**a**–**g**) AFM images of a VP flake undergoing 30 min exposure in ambient condition, immediately after 100 μW 532 nm laser illumination, and 200 °C N_2_ annealing for 5 h and 10 h, respectively. The embedded figures in (**a**,**c**) show the corresponding OM images. The inset curves are the height profiles along the green dashed lines. (**b**–**d**) Magnified AFM images of (**a**–**g**). Insets show the height profiles of the bumps in (**f**,**h**). (**i**) AFM map of the scanning region disclosed by the green dashed rectangle in (**h**); the inset presents the height profile along the hole. (**j**) SEM image of the region in (**i**). (**k**) Height distribution of the VP flake surface in different states. (**l**) Corresponding roughness of (**k**).

While the natural degradation rate of VP in ambient conditions is relatively slow in a time scale of hours, it is found that laser illumination can considerably accelerate the VP degradation process. As depicted in Figure S3, following 100 μW 532 nm laser illumination in the air for mere tens of seconds, evident bumps and holes appear at the illuminated positions, denoted by the orange arrows in Figure S3c, which reflect a degradation speed substantially faster than that without laser illumination. To investigate the photodegradation-induced morphological variations, we conducted systematic atomic-force microscopy (AFM) measurements on the VP flake displayed in Figure S3. Figure 2a,b present the AFM images of this VP flake undergoing 30 min of exposure in ambient conditions following exfoliation. The VP flake demonstrated an average height of 125.6 nm. Numerous tiny adsorbed droplets were observed across the VP surface, which can be assigned to the aforementioned hydrophilicity of VP. Subsequently, 100 μW 532 nm laser illumination was applied to specific positions on the VP surface, with the illumination duration (tens of seconds) differing at various positions in ambient conditions. Figure 2c,d show that more adsorbates emerged on the surface and aggregated into broader and higher formations after laser illumination. As a result, the average height of the VP surface rose to 140.7 nm. Concurrently, the surface roughness witnessed a significant increase after laser illumination, as evident from the considerable broadening of the height distribution and the elevation in the root mean square (RMS) values in Figure 2k,l, respectively.

The presence of this light-induced adsorbate film covering the majority of the VP surface posed challenges for further investigating the structural variations beneath. To overcome this obstacle, we employed nitrogen thermal annealing of the sample in a tube furnace operating under flowing nitrogen gas at 200 °C for 5 h. As a consequence, the average height of the VP flake decreased to 128.3 nm, accompanied by a dramatic improvement in the RMS to 9.879 nm (Figure 2l), comparable to the values before laser illumination. Meanwhile, the height distribution of the VP surface became significantly narrower (Figure 2k), indicating an excellent surface-cleaning effect achieved by thermal annealing. As presented in Figure 2e,f, most of the adsorbates were successfully removed, restoring a clean and uniform surface across most areas. However, the laser-illuminated positions became visible, resembling emerging reefs after a receding tide. This is possibly because defects with dangling bonds were produced at these laser-illuminated positions, which was more favorable for HPO_x_ accumulation than at the rest area.

Additionally, it was found that longer illumination durations led to increased adsorbate accumulation, which was manifested by the higher bump at point 2 (illumination duration 90s) compared with point 1 (60 s), as shown in Figure 2f. In order to study the intrinsic morphological details of the illuminated positions, an additional nitrogen thermal-annealing step (flowing nitrogen gas, 200 °C, 5 h) was performed on this sample. As illustrated in Figure 2g,h, the average height of the VP surface remained nearly unchanged, while the RMS slightly improved from 9.879 nm to 7.09 nm (Figure 2l). Importantly, with the elimination of the adsorbate bump, the intrinsic morphology at these illuminated positions became unveiled. According to the height profiles shown by the insets in Figure 2f,h, the height of the bump at point 2 dramatically dropped from 350 nm to 81 nm after the second thermal-annealing step. Regarding point 1, as presented by the AFM and SEM images in Figure 2i,j, a circle-shaped hole with a diameter of approximately 600 nm and a depth of about 59.86 nm was observed, demonstrating a prominent etching effect induced by laser illumination. Moreover, with the benefit of the tiny size of the laser spot (~0.5λ) and rapid photodegradation rate, laser illumination may emerge as an applicable method for high-resolution and efficient selected-area etching on VP, which is further reinforced by the scanning etching conducted on the VP flake in Figure S4.

**Figure 3:**
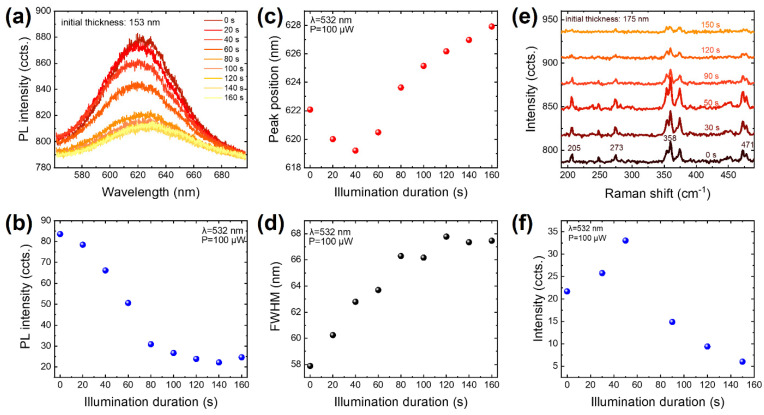
Laser illumination-duration-dependent Raman and photoluminescence. (**a**) PL spectra evolution of a VP flake (initial thickness: 153 nm) at different laser illumination durations. (**b**–**d**) Intensity, peak position, and FWHM of PL spectra at various illumination durations. (**e**) Raman spectra evolution of another VP flake with an initial thickness of 175 nm at different laser illumination durations. Four representative Raman modes are labeled. (**f**) Raman intensity at various illumination durations. The laser wavelength is 532 nm, with an excitation power of 100 μW.

Raman and photoluminescence spectroscopy are effective and universally used characterization tools for layer-thickness identification [37,38,39], doping analysis [37,40,41,42,43], and defect detection [44] of nanomaterials. According to this, we conducted PL and Raman measurements on VP flakes at various illumination durations. The corresponding illumination-duration-resolved data are presented in Figure 3. Figure 3a exhibits the PL spectra of an initial 153 nm thick VP at various illumination durations, revealing a clear dependence of PL on illumination duration. Specifically, as plotted in Figure 3b, the relative PL intensity of the VP sample declined from approximately 83 to around 25 when the illumination duration increased from 0 s to 160 s. At the same time, with increasing illumination duration, the full width at half maximum (FWHM) increased monotonically, as shown in Figure 3d. What differed was that the peak position initially displayed a blueshift from about 622 nm to approximately 619 nm until the illumination duration of 40 s, then it underwent a redshift and ultimately reached approximately 628 nm, as shown in Figure 3c. The observed trends in PL intensity and FWHM evolution may result from illumination-induced defects and thickness thinning of the VP. Since the optical bandgap of VP is thickness-dependent, thickness thinning would modify its optical bandgap, while defects can introduce new energy states and levels, both potentially leading to PL-intensity quenching and FWHM broadening.

In addition to the photoluminescence spectra, the Raman spectra also exhibit a clear dependence on illumination duration. As plotted in Figure 3e, several prominent Raman peaks, including those at 205, 273, 358, and 471 cm^−1^, are observed in a VP sample whose initial thickness is 175 nm. All four peaks evolved over the illumination duration, but for clarity, only the 358 cm^−1^ peak with the highest signal-to-noise ratio is discussed here. This peak originates from the stretching mode for [P8] cages [45], and its intensity evolution with respect to the illumination duration is depicted in Figure 3f. With increasing illumination duration, its intensity initially rose, showing an inflection point at 50 s, and then fell until the end of this examination, ultimately reaching a level considerably lower than that of the pristine state. In contrast, no noticeable peak shift is observed with various illumination durations.

As a comparative analysis, Raman and PL examinations were conducted on multiple individual VP flakes possessing distinct layer thicknesses, and the corresponding data are presented in Figure S5. Generally speaking, both the PL and Raman spectra demonstrate an apparent thickness dependence. As the layer thickness reduces, the PL intensity decreases monotonically, as shown in Figure S5b. Additionally, as plotted in Figure S5c, the PL peak position initially shifts to lower wavelengths from around 645 nm (for the 175 nm thick VP flake) to about 606 nm at a thickness of 51 nm, and then turns to shift upwards to ~635 nm at a thickness of 26 nm. Regarding the Raman variations demonstrated in Figure S5e,f, the Raman intensity first rises to a maximum as the thickness decreases from 175 nm to 51 nm and then turns to decrease as the thickness further reduces. However, no evident relation was observed between the FWHM of the PL and layer thickness in Figure S5d. Interestingly, the trends of PL intensity, PL peak shift, and Raman intensity in response to the thickness decline exhibit similarities to their counterparts in response to the illumination duration. This provides further validation of the laser-illumination-induced etching effect on VP from a different perspective. For the morphology and thickness identifications, the OM and AFM images of the VP samples with distinct thicknesses used in Figure 3 and Figure S5 are provided in Figure S6.

**Figure 4:**
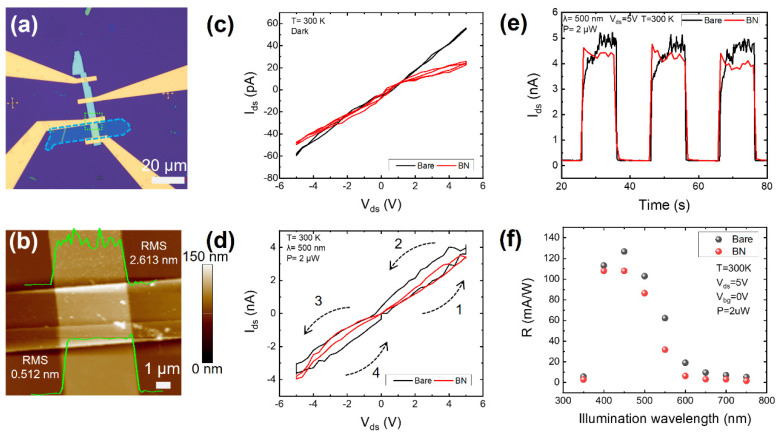
Electrical and optoelectrical characteristics of naked and BN-passivated VP. (**a**) OM image of the half-passivated VP device. The blue dashed lines outline the BN-passivated region. (**b**) AFM map of the region disclosed by the green dashed rectangle in (**a**). The insets show the height profiles of the BN-passivated and naked channels, and the corresponding RMS values are indicated. (**c**) Electrical-transport properties of bare (black) and BN-passivated (red) channels under dark conditions. (**d**) I-V curves of bare and BN-passivated channels under 500 nm xenon lamp illumination. The black dashed arrows labeled by serial numbers denote the bias scanning sequence. (**e**) Photoswitching curves of bare and BN-passivated channels. (**f**) The spectral responsivity of bare and BN-passivated channels.

In an attempt to gain further insight into the photodegradation mechanism, study its impact on the electrical and optoelectrical properties of VP, and explore the passivation method against the degradation of VP, as shown in Figure 4a, we transferred a 19 nm thick hexagonal boron nitride (hBN) flake (blue dashed line outlined in Figure 4a) to cover a partial region of a VP flake on a silicon oxide substrate. The mechanical exfoliation and transfer steps were performed in a glove box to prevent H_2_O/O_2_ adsorption prior to the electrical and optoelectrical examination. Subsequently, this partially passivated VP sample was fabricated into a photodetector with electrodes (5/70 nm Cr/Au). The BN-covering area of the VP serves as the passivated channel, while the remaining area functions as the bare channel. The AFM image in Figure 4b reveals a clean and uniform interface between the hBN and VP. In contrast, there existed numerous adsorbates across the bare VP channel, rendering a rougher surface with a higher RMS value of 2.613 nm compared with the 0.512 nm of the hBN-passivated channel. It should be noted here that the VP device underwent a brief exposure to air after fabrication and before examination, explaining the presence of the adsorbates distributed across the bare channel. Interestingly, prior to the AFM, electrical, and optoelectrical measurements, we employed 532 nm laser illumination with a high power of 1 mW for a long duration of 5 min on the hBN-passivated channel. However, as the OM and AFM images demonstrated, no evident degradation-induced bumps or holes were observed. Considering the experimental observations above, it can be concluded that van der Waals (vdW) passivation with hBN can effectively exclude H_2_O/O_2_ adsorbates and the resultant degradation from the VP surface underneath.

Furthermore, we conducted electrical and optoelectrical measurements on the as-fabricated VP device. Figure 4c presents the electrical-transport properties of the bare (black) and hBN-passivated (red) channels under dark conditions; it seems that the bare channel demonstrates a higher current than the BN-passivated channel at an identical bias. This indicates a lower resistance for the bare channel, given the same channel width and length. This difference might arise from the adsorbate-doping-induced carrier density improvement within the bare channel [46,47,48]. Upon illumination, as shown in Figure S7a,c, both the bare and hBN-passivated channels demonstrate a prominent photoresponse within the visible range (350~750 nm). Notably, both the bare and hBN-passivated channels show photocurrent hysteresis with a scanning bias. Nevertheless, the hysteresis is more pronounced for the bare channel across all examined illumination wavelengths, as exemplified by the I-V curves illuminated by 500 nm that is plotted in Figure 4d. This observed hysteresis discrepancy between the bare and hBN-passivated channels can also be interpreted due to adsorbate-induced effects [49]. The adsorbates on the bare channel could trap photogenerated carriers of one polarity, leaving behind photocarriers with the opposite polarity to transport across the channel under an applied bias. As the bias increases forward from 0 to ±5 V (steps 1 and 3 labeled in Figure 4d, respectively), the carrier in the channel would transport at an increasingly faster speed, debilitating the photocarrier recombination and thus improving the photogain, as reflected by the increasing photoconductance. Conversely, as the bias decreases backward from ±5 V to 0 (steps 2 and 4, respectively), the increasing carrier transit time in the channel would boost the photocarrier recombination, weakening the photogain, signified by a decreasing photoconductance. Therefore, the bare channel holding more adsorbates would readily demonstrate a more noticeable photocurrent hysteresis in comparison with the hBN-passivated channel.

In addition, the photoswitching dynamics of the VP device were investigated, and the results are provided in Figure 4e and Figure S7b,d. The on-state photocurrent of the bare channel is slightly higher than that of the hBN-passivated channel at the illumination wavelength of 500 nm (in Figure 4e) and other illumination wavelengths (Figure S7). Importantly, from Figure S7b,d, we can see that for illumination wavelengths ranging from 350 nm to 750 nm, the bare and hBN-passivated channels demonstrate different time-resolved photoresponse characteristics. Specifically, the current of the hBN-passivated channel rises quickly and directly to a relatively plane plateau when illuminated. In comparison, the current of the bare channel shows a two-stage photoresponse. Initially, the current of the bare channel rises rapidly during the first stage upon illumination; then it continues rising, but at a much slower speed, during the second stage. While the quick-response stage observed in both the bare and hBN-passivated channels is determined by the photoconductive response, the slow-response stage uniquely observed in the bare channel potentially results from the bolometric effect [50,51] or trap-state effect [52], according to the relevant literature. In our study, both the bare and hBN-passivated channels consist of the same violet phosphorus flake with identical geometry; hence, it is unlikely for the bolometric effect to exclusively manifest in the bare channel while being absent in the hBN-passivated channel. In other words, it is the adsorbates on the bare channel surface that would trap photogenerated carriers, prolong their lifetime, and consequently give rise to a slow photoresponse stage. Also, by calculating the responsivity of both the bare and hBN-passivated channels for all examined illumination wavelengths, the spectral responsivity was obtained. As plotted in Figure 4f, the bare channel shows minor responsivity enhancements compared with that of the hBN-passivated channel for all examined illumination wavelengths, which confirms the contribution of the adsorbate-induced trap state to the photogain improvement.

## 4. Discussion

**Figure 5:**
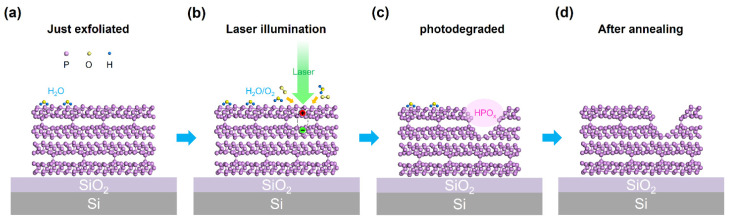
Schematic illustration of the photodegradation and annealing effects on a VP flake. (**a**–**c**) The VP flake in different states. (**a**) The VP flake that has just been exfoliated onto a silicon oxide substrate, with H_2_O molecules adsorbed on its surface after an instant exposure to the air; atoms of different elements are distinguished by various colors and sizes. (**b**) The VP flake undergoing 532 nm laser illumination. (**c**) The VP flake after photodegradation. (**d**) The VP flake after nitrogen-gas thermal annealing.

According to the experimental results above, we can make some conclusions regarding the underlying mechanism of VP degradation and surface cleaning effects by annealing, as shown in Figure 5. In ambient conditions, the hydrophilic nature of the VP surface facilitates the adsorption of H_2_O/O_2_. These adsorbed H_2_O/O_2_ molecules subsequently react with phosphorus in the VP lattice to form PO_x_ (Figure 5a), thus leading to the degradation of VP at a relatively slow rate. However, when illuminated by a laser with a photon energy above the VP bandgap, the degradation speed is dramatically enhanced. The accelerated degradation, induced by 532 nm laser illumination, is graphically illustrated in Figure 5b. During laser illumination, excitons are generated on the surface at the illuminated positions, acting as adsorption centers for H_2_O/O_2_ from the air due to their dipole momentum. After that, the light-induced excitons transfer charge carriers to the aqueous O_2_, producing intermediate superoxide O_2_^−^ [23], which reacts with the phosphorus surface to form phosphorus oxide (PO_x_). As illustrated in Figure 5c, this PO_x_ readily transforms into aqueous phosphate species (HPO_x_) in the presence of adsorbed H_2_O [53], preventing the oxide accumulation and mitigating the decrease in reaction speed. Since there exist surface defects with dangling bonds at the illumination points, the reaction product, HPO_x_, tends to accumulate at these locations [12,54], explaining where the visible bumps on the VP surface come from. Also, owing to the efficient generation of reactive oxygen species by light-induced excitons, VP demonstrates a significantly faster degradation rate compared with that without laser illumination. It is noteworthy that we have also employed 1064 nm laser illumination with a considerably high power of 20 mW and a long duration of 5 min on VP flakes; however, no evident degradation was observed at the 1064 nm laser-illuminated positions. We attribute this to the fact that the photon energy of the 1064 nm laser is lower than that of 532 nm laser, and, crucially, the photon energy of the 1064 nm laser is below the optical bandgap of violet phosphorus. Consequently, the generation of excitons and photocarriers would be significantly reduced, which further debilitates the degradation reaction. Additionally, as shown by Figure 5d, undergoing thermal annealing (in flowing nitrogen gas, 200 °C) would evaporate the adsorbed H_2_O/O_2_ on the VP surface, hence revealing the intrinsic surface of VP. In addition, the passivation of VP with hBN enhances its stability by creating atomically fitting interfaces that effectively exclude the adsorbed H_2_O/O_2_, which consequently prevents degradation. Without the involvement of H_2_O and O_2_, degradation does not occur in the hBN-passivated regions of VP, even under light illumination. In other words, we hypothesize that there are two essential factors required for the degradation of VP, adsorbed H_2_O and adsorbed O_2_, while the illumination with a photon energy higher than the optical bandgap of VP would significantly accelerate the degradation process.

## 5. Conclusions

To summarize, we have experimentally demonstrated the photo-accelerated degradation of VP through morphological and spectroscopic characterization. Utilizing N_2_ thermal annealing on the photodegraded samples revealed the intrinsic morphological details at the illumination points and showcased a significant etching effect induced by laser illumination, which provides a possible method for the efficient selected-area etching of VP at high resolution. Additionally, van der Waals passivation of VP was achieved by transferring a 2D hBN flake onto it, effectively excluding absorbates and preventing photodegradation, as validated by the distinct electrical and optoelectrical characteristics compared with the bare counterpart. Overall, our work enhances the comprehension of VP degradation, unveils the light-illumination impact on accelerating the degradation, and provides a simple but effective method to passivate VP, laying the groundwork for emerging fundamental and application-oriented studies. The findings and methods showcased in this work can be readily applied to a broad spectrum of volatile materials, showing promise for the continued exploration of innovative avenues in the captivating field of low-dimensional electronics.

## Data Availability

Data are contained within the article and supplementary materials.

## Supplementary Materials

The following are available online at https://www.mdpi.com/article/10.3390/nano14050422/s1, Figure S1: Air-exposure-duration-dependent degradation of an exfoliated VP flake; Figure S2: SEM images of a VP flake after various exposure time in ambient conditions; Table S1: Elemental-concentration change in a bulk VP crystal with increasing exposure duration in ambient conditions; Figure S3: OM images of photo-accelerated degradation in a VP flake; Figure S4: Scanning etching on a VP flake by photodegradation; Figure S5: Thickness-dependent Raman and photoluminescence of VP; Figure S6: Thickness identifications of VP flakes; Figure S7: Optoelectrical characteristics of bare and BN-passivated VP channels at various illumination wavelengths.
