# Supplementary material for: Photodegradation and van der Waals Passivation of Violet Phosphorus
Source: Nanomaterials (Basel). 2024 Feb 25;14(5):422. doi: 10.3390/nano14050422 (PMC10935355; doi:10.3390/nano14050422)
Supplement: Supplementary file 1 [file nanomaterials-14-00422-s001.zip › nanomaterials-2846056-supplementary.pdf]

Supporting Information

Photodegradation and van der Waals Passivation of Violet Phosphorus

iangzhe Zhang [1], Bowen Lv [1], Haitao Wei [1], Xingheng Yan [2], Gang Peng [3,*] and Shiqiao Qin [1,*]

1 *College of Advanced Interdisciplinary Studies, National University of Defense Technology, Changsha 410073, China*

2 *College of Aerospace Science and Engineering, National University of Defense Technology, Changsha 410073, China*

3 *College of Science, National University of Defense Technology, Changsha 410073, China*

* *Correspondence: penggang@nudt.edu.cn (G.P.); sqqin8@nudt.edu.cn (S.Q.)*

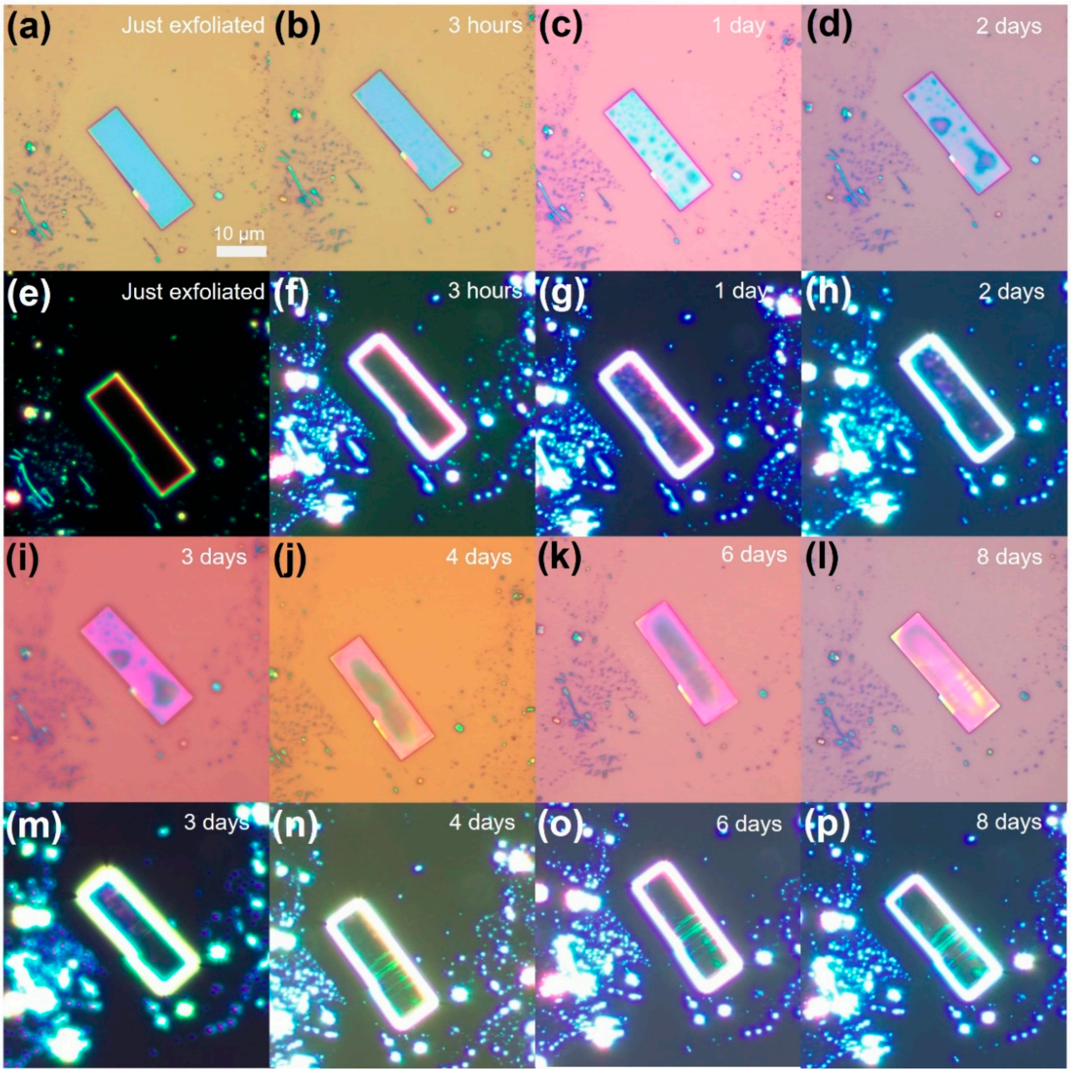

FIG. S1. Air exposure duration-dependent degradation of an exfoliated VP flake. (a-d) & (i-l) Bright-field optical micrographs of the VP flake after different air exposure time. (e-h) & (m-p) The corresponding dark-field optical micrographs.

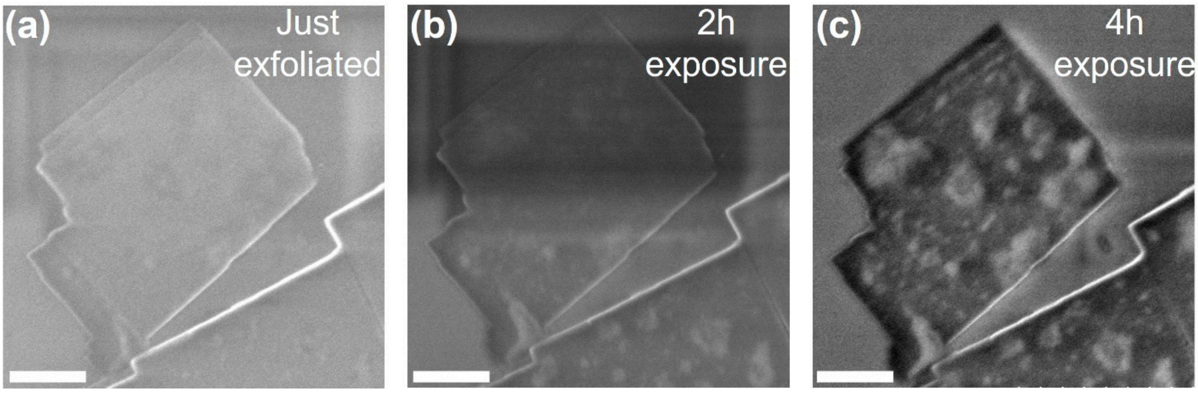

FIG. S2. SEM images of a VP flake after various exposure time in ambient conditions. (a) Just exfoliated. (b) 2 hours exposure. (c) 4 hours exposure. The scale bar is 500 nm.

	pristine	1 day	2 day
P(2p)	45.03%	33.18%	29.94%
O(1s)	54.97%	66.82%	70.06%

Table S1. Elemental concentration change of a bulk VP crystal with increasing exposure duration in ambient conditions.

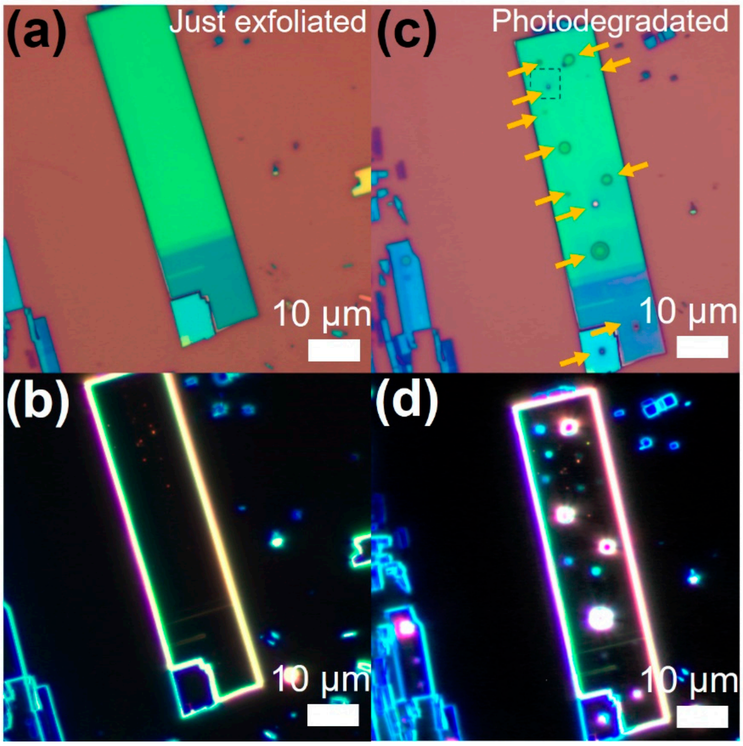

FIG. S3. OM images of photo-accelerated degradation in a VP flake. (e) & (f) just exfoliated. (g) & (h) after 532 nm laser illumination. The orange arrows in (g) denote the laser illumination points.

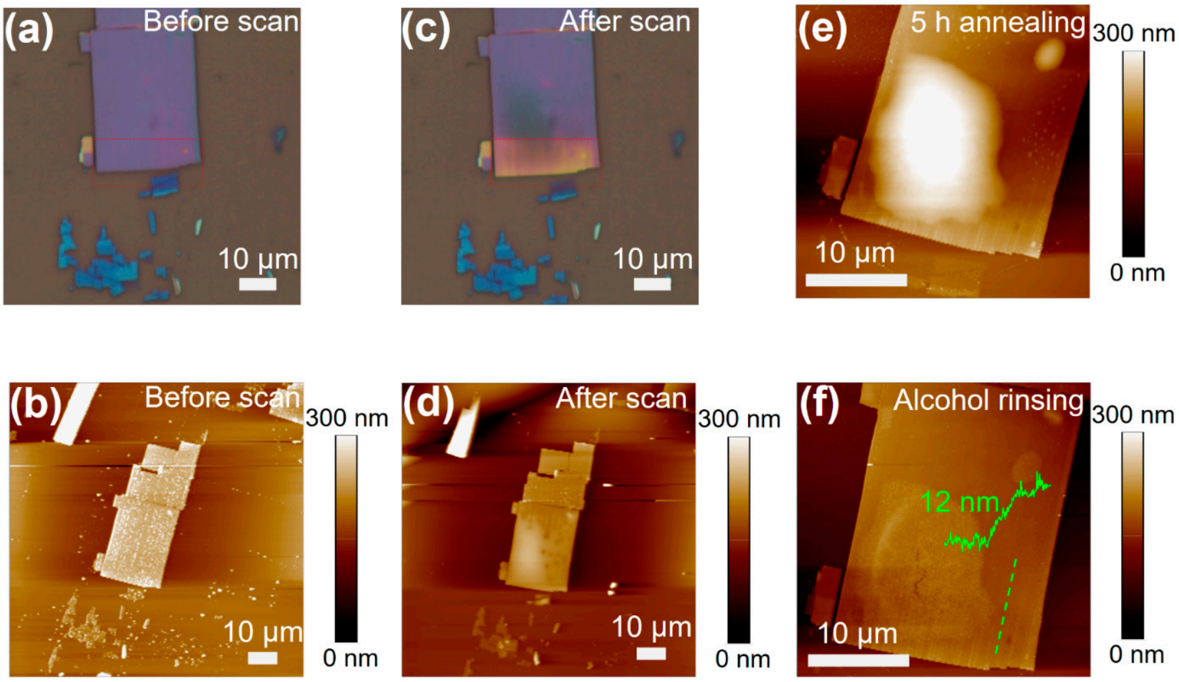

FIG. S4. Scanning etching on a VP flake by photodegradation. (a) & (b) OM and AFM images before laser scan. (c) & (d) OM and AFM images after laser scan. (e) & (f) AFM images of the VP flake successively after 5 hours of 200 °C N_2 annealing and alcohol rinsing, respectively. The inset in (f) shows height profiles along the green dashed line.

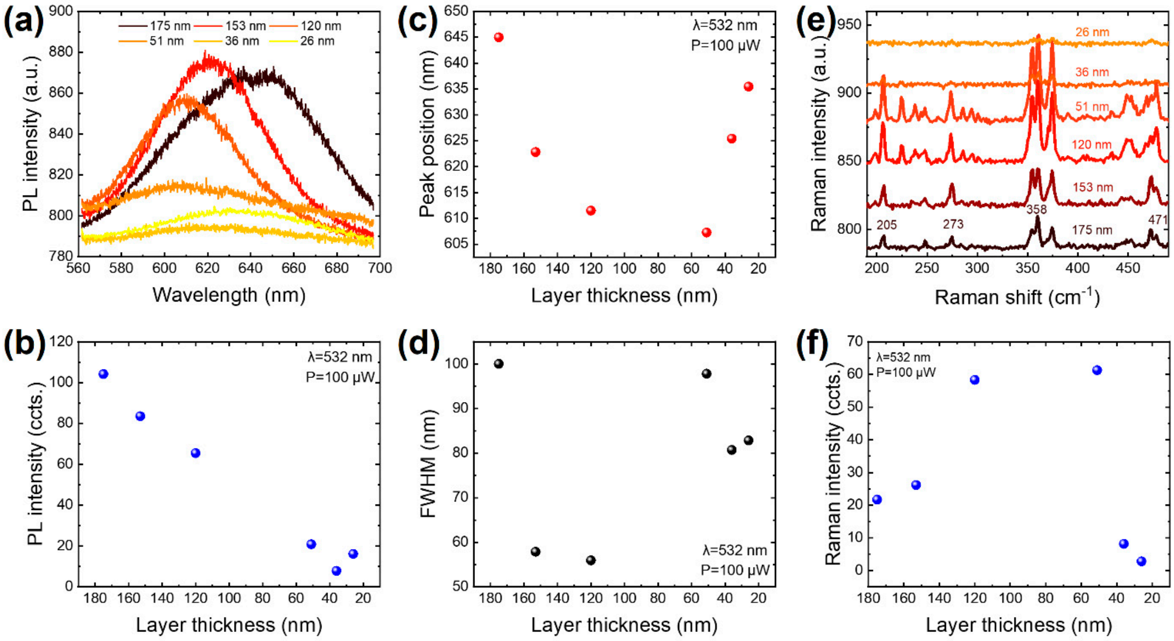

FIG. S5. Thickness-dependent Raman and photoluminescence. (a) PL spectra evolution of VP flakes with different thicknesses. (b-d) Intensity, peak position, and full width at half maximum (FWHM) of PL spectra with various thicknesses. (e) Raman spectra evolution with various thicknesses. Four representative Raman modes are labeled. (f) Raman intensity at various thicknesses. The wavelength and power of the laser are 532 nm and 100 μW, respectively.

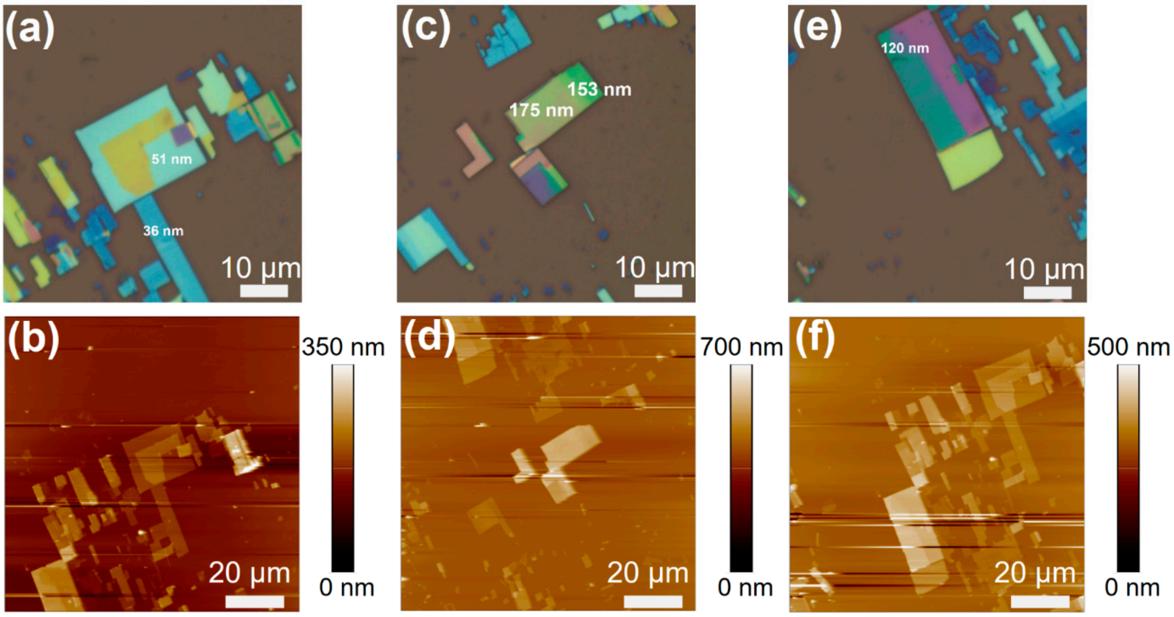

FIG. S6. Thickness identifications of the VP flakes in Figure S3. (a) & (c) & (e) OM images of VP flakes. Thickness values are labeled on corresponding locations. (b) & (d) & (f) Corresponding AFM images.

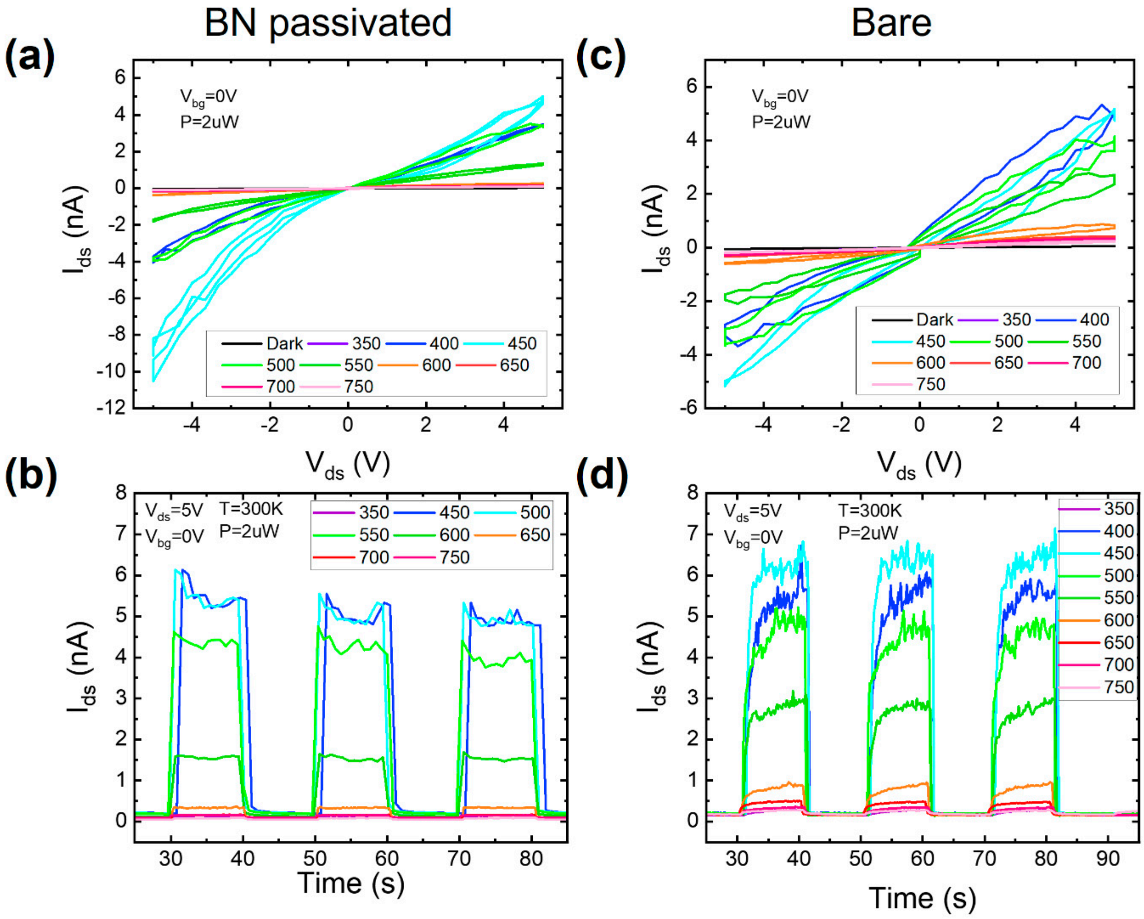

FIG. S7. Optoelectrical characteristics of bare and BN passivated VP channels. Photoresponse properties of BN passivated (a) and bare channels (c) under different illumination wavelengths. Photoswitching dynamics of BN passivated (b) and bare channels (d).
